# Long-Time Treatment by Low-Dose *N*-Acetyl-L-Cysteine Enhances Proinflammatory Cytokine Expressions in LPS-Stimulated Macrophages

**DOI:** 10.1371/journal.pone.0087229

**Published:** 2014-02-04

**Authors:** Tomokazu Ohnishi, Kenjiro Bandow, Kyoko Kakimoto, Joji Kusuyama, Tetsuya Matsuguchi

**Affiliations:** Department of Oral Biochemistry, Kagoshima University Graduate School of Medical and Dental Sciences, Kagoshima, Japan; Temple University School of Medicine, United States of America

## Abstract

*N*-acetyl-L-cysteine is known to act as a reactive oxygen species scavenger and used in clinical applications. Previous reports have shown that high-dose *N*-acetyl-L-cysteine treatment inhibits the expression of proinflammatory cytokines in activated macrophages. Here, we have found that long-time *N*-acetyl-L-cysteine treatment at low-concentration increases phosphorylation of extracellular signal-regulated kinase 1/2 and AKT, which are essential for the induction of proinflammatory cytokines including interleukin 1β and interleukin 6 in lipopolysaccharide-stimulated RAW264.7 cells. Furthermore, long-time *N*-acetyl-L-cysteine treatment decreases expressions of protein phosphatases, catalytic subunit of protein phosphatase-2A and dual specificity phosphatase 1. On the other hand, we have found that short-time *N*-acetyl-L-cysteine treatment at low dose increases p53 expression, which inhibits expressions of proinflammatory cytokines. These observations suggest that long-time low-dose *N*-acetyl-L-cysteine treatment increases expressions of proinflammatory cytokines through enhancement of kinase phosphorylation.

## Introduction

Reactive oxygen species (ROS) are involved in various cellular events including cell proliferation, apoptosis and immune responses [1], [2]. *N*-acetyl-L-cysteine (NAC) is a precursor of L-cysteine, which is converted to intracellular gluthathione acting as a scavenger of ROS [3]. NAC has been clinically used in the treatment of acetoaminophen overdose to reduce liver injury for more than 30 years [4]. Recent studies have revealed more beneficial effects of NAC on various diseases including cancer, heart disorders, chronic obstructive pulmonary disease (COPD), human immunodeficiency virus (HIV) infection, diabetes-associated periodontal bone loss and contrast-induced nephropathy [3], [5], [6].

Macrophages are vital for the recognition and elimination of microbial pathogens. They are critical immune effecter cells, which are associated with not only antigen-specific immune responses but also innate immune responses [7]. Lipopolysaccharide (LPS), which is the endotoxin of gram-negative bacteria, is a strong stimulator for the innate immune response. LPS activates macrophages through the Toll-like receptor (TLR) 4 signal pathway to induce gene expressions of proinflammatory cytokines. Interaction of LPS with TLR4 leads to the activation of both nuclear factor-κB (NF-κB) and MAPK cascades, which are mediated by MyD88- and TRIF-dependent pathways [8]–[10]. The LPS/TLR4 signal induces activation of IκB kinase (IKK), and phosphorylation and degradation of IκBα, which masks the nuclear localization signal of NF-κB [9]. The degradation of IκBα causes nuclear translocation of NF-κB, which induces gene transcriptions of various proinflammatory cytokines including IL-1β and IL-6 [9]. On the other hand, cascades of MAPKs, including JNKs, ERK1/2 and p38, are also activated by the LPS/TLR4 signal through both MyD88- and TRIF-dependent pathways to phosphorylate activator protein-1 (AP-1) transcription factors [8]–[10]. Cancer Osaka thyroid oncogene/tumor progression locus 2 (Cot/Tpl2) kinase is a downstream mediator of TLR4 signal stimulating MAPK activation [11]. MAPK activation leads to nuclear translocation of AP-1, which interacts with the AP-1-binding sites in gene promoters of various proinflammatory cytokines to induce promoter activities [8]–[10]. Gene promoters for IL-1β and IL-6 contain binding motifs for NF-κB and AP-1, both of which are essential for their expressions in response to LPS.

In innate immune responses, macrophages activate NADPH oxidase, which produces ROS to eliminate microbial pathogens as well as infected cells, and to increase production of proinflammatory cytokines [8]–[10], [12]. Several previous reports have shown that in vitro NAC administration is inhibitory to the ROS-mediated apoptosis and innate immune responses of macrophages [13]–[17]. Most of these studies examined the negative regulatory effects of NAC at concentrations higher than 10 mM on innate immune responses such as expressions of proinflammatory cytokines [13]–[17]. In contrast, NAC treatment of LPS-stimulated macrophages at a low-concentration (2.5 mM) was reported to increase expression of IL-12, an essential cytokine for the induction and maintenance of helper T type 1 (Th1) cell development [18]. Notably, according to several recent clinical studies, symptoms of COPD and psychiatry diseases were improved by oral administration of NAC at doses lower than those for acetoaminophen intoxication [4], [19], [20]. The dose of NAC clinically used for the treatment of acetoaminophen intoxication has not been significantly changed for more than 30 years. In addition, intravenous administration of NAC to patients with acute sever sepsis is reported to aggravate sepsis-induced organ failure, suggesting that the effects of NAC are not always anti-inflammatory [21]. Therefore, it is important to examine the effects of low-dose NAC treatment on innate immune responses such as expressions of proinflammatory cytokines.

High-dose NAC treatment has been reported to inhibit expressions of proinflammatory cytokines in LPS-stimulated macrophages, while it reduces LPS-induced phosphorylation of AKT and IKKα/β [13]. NF-κB is an important downstream target of phosphatidylinositol-3 kinase (PI3K)/AKT [22]. AKT binds to and increases the activity of IKKα, which is required for NF-κB activation [23]. Consequently, high-dose NAC treatment reduces promoter activity containing binding motif for NF-κB [23]. Recently, AP-1 has also been shown to be a downstream target of AKT/IKKα [24]. Consistently, a mutation of AP-1 site in IL-6 gene promoter, but not the NF-κB site, is reported to reduce the induction of transcriptional activity of IL-1β [24]. These observations suggest that both transcription factors, NF-κB and AP-1, are downstream targets of AKT.

In this study, we have revealed that low-dose NAC treatment for long periods enhance expressions of pro-inflammatory cytokines, interleukin 1β (IL-1β) and interleukin 6 (IL-6), in LPS-stimulated macrophages. When cells are treated with low-dose NAC for long periods, LPS-induced phosphorylation of AKT and ERKs are enhanced, which are responsible for the increased promoter activities of IL-1β and IL-6 genes through activation of AP-1 transcription factor. Furthermore, our data have also revealed that the increased expression of a tumor suppressor protein, p53, is associated with negative regulation of proinflammatory cytokine expressions by low-dose NAC treatment for short-time.

## Materials and Methods

### Reagents and Antibodies

NAC and LPS from *E. coli* were purchased from Sigma-Aldrich (St Louis, MO). SP600125, a specific JNK inhibitor was purchased from BIOMOL International (Plymouth Meeting, PA). U0126, a specific inhibitor of ERK activation pathway, SB203580, a specific p38 kinase inhibitor, SP 600125, a specific JNK inhibitor and LY294002, and a specific inhibitor of AKT phosphorylation were from CALBIOCHEM (San Diego, CA). Antibodies specifically recognizing phosphorylated forms of JNKs, ERKs, p38 kinases, and AKT were purchased from Cell Signaling Technology (Danvers, MA). Antibodies against ERK1/2, JNK1/2, JNK2, AKT, and p38 kinases, were also from Cell Signaling Technology. Antibodies against p53 and GAPDH were purchased from Santa Cruz Biotechnology (Santa Cruz, CA).

### Cell Culture

RAW264.7 cells (ATCC TIB71, Manassas, VA) were cultured in 10% fetal calf serum (FCS)-containing Dulbecco’s Modified Eagle Medium (DMEM) as previously described [25]. RAW264.7 cells were pretreated with 2 mM or 20 mM NAC, and then stimulated with 100 ng/ml LPS for 3 hours. Peripheral blood mononuclear cells (PBMCs) were isolated from human blood with Lymphoprep™ (Axis-Shield ProC AS, Oslo, Norway) following the protocol for Lymphoprep™. The PBMCs were plated in a 12 well dish coated with poly-L-lysine, and then incubated in RPMI plus 10% FCS at 37°C for 12 hours. After washes with PBS, the cells were incubated in RPMI1640 plus 10% FCS containing 50 ng/ml human macrophage-colony stimulating factor (M-CSF) (Kyowa Hakko Bio Co. Ltd, Tokyo, Japan) for 3 days to induce the differentiation of macrophages. The cells in some wells were pretreated with NAC at a concentration of 2 mM or 20 mM, and then stimulated with 100 ng/ml LPS.

### Tetracycline-inducible (Tet-On) Expression System for p53

A TetOn inducible expression system for mouse p53 was established as previously described [26]. The coding region of the mouse p53 cDNA was amplified by reverse transcription-PCR (RT-PCR) using a pair of primers, tp53/F158: ggatccatgactgccatggaggagtc, and tp53/1321R: gcggccgcagaggcagtcagtctgagtc, from the total RNA isolated from RAW264.7 cells. The 1.2 kb BamHI and NotI- digested fragment was inserted into pTRE2Hyg plasmid (Clontech, CA). The constructed pTRE2Hyg-tp53 was stably transfected into RAW264.7 pEF-1α-pTet-On cell line. The hygromycin B-selected clones were treated with or without 2 µg/ml doxycycline for 2 days, and protein and total RNA were extracted from cells as described below.

### Luciferase Reporter Gene Assay

RAW264.7 cells stably transfected with pNFκB-Luc or pAP-1-Luc plasmid (Clontech) were prepared by co-transfection with pcDNA3.1(+) and selection with 1 mg/ml G418. After pre-treatment with 2 mM NAC, the cells were stimulated with 100 ng/ml LPS for 6 hours. Luciferase activity was measured by Luciferase assay systems (Promega, Corp, WI) according to the manufacturer’s instructions.

The promoter region (−254 to +23) of the mouse IL-1β gene containing the putative transcriptional initiation site was cloned by PCR from C57BL/6 genomic DNA using a pair of primers, CCAGATGAGCCTATTAGGCC and TCCACCACGATGACACACTT, and ligated into the luciferase reporter vector pGV-p (Toyoink, Tokyo, Japan) to generate pGV-p-IL1β pGV-p-IL1β or the vector control, pGV-p was transfected into 70% confluent RAW264.7 pEF-1α-pTet-On pTRE2Hyg-p53 cell line using Lipofectamine 2000 (Invitrogen), according to the manufacturer’s instructions. At 24 hours after transfection, the cells were treated with 2 µg/ml doxycycline and further incubated for 48 hours. Luciferase activities were measured and normalized by protein contents of the cell lysates.

### Oligonucleotides

Amplicons and the corresponding oligonucleotides were as follows: tp53 (tp53/141F: 5′gcttctccgaagactggatg3′, tp53/283R: 5′gtccatgcagtgaggtgatg3′: NM_001127233.1), PP2Ac (catalytic subunit of protein phosphatase 2A) (PP2Ac/F: 5′ ctctcactgccttggtggat 3′, PP2Ac/R: 5′tgaccacagcaagtcacaca 3′, NM_019411), DUSP (dual specificity phosphatase) 1 (DUSP1/F: 5′ atccctgtggaggacaacc3′, DUSP1/R: 5′ gaggtaagcaaggcagatgg 3′; NM_013642), human IL-1β (hIL-1β/L: 5′ gtggcaatgaggatgacttgt 3′, hIL-1β/R: 5′ ctggaaggagcacttcatctg 3′, NM_000576), human IL-6 (hIL-6/F: 5′ cagacagccactcacctcttc 3′, hIL-6/R: 5′ ttcaggttgttttctgccagt 3′, NM_000600), and human ubiqutin C.(hUbc/F: 5′ atttgggtcgcagttcttgtt 3′, hUbc/R: 5′ ttgtcaagtgacgatcacagc 3′, NM_021009).

### Real Time RT-PCR and Western Blot Analysis

Total RNA was extracted as previously described [11]. Each real-time RT-PCR reaction was run in triplicate and normalized against Ubc mRNA level. For western blot analyses, total cellular lysate preparation and immunoblotting procedures with specific antibodies were performed as previously described [27].

### Measurement of ROS in RAW264.7 Cells

RAW264.7 cells were stimulated with or without 100 ng/ml LPS in Hank’s Balanced Salt Solution (HBSS) containing 20 µM 2′,7′-dichlorodihydrofluorescein diacetate (H_2_DCF-DA) for 1 hour. The cells were washed with PBS, and then the fluorescence emission of the cell suspensions in PBS was assessed by Fluoroskan Ascent Microplate Fluorometer (Thermo Fisher Scientific Inc, Waltham, MA).

### Statistical Analysis

The values given are mean S.E.M. Statistical analysis between two samples was performed using student’s t-test. In all cases, *P*<0.05 was considered as being significant.

## Results

### Effects of Low-dose NAC Treatment on Proinflammatory Cytokine Expressions in Macrophages

**Figure 1:**
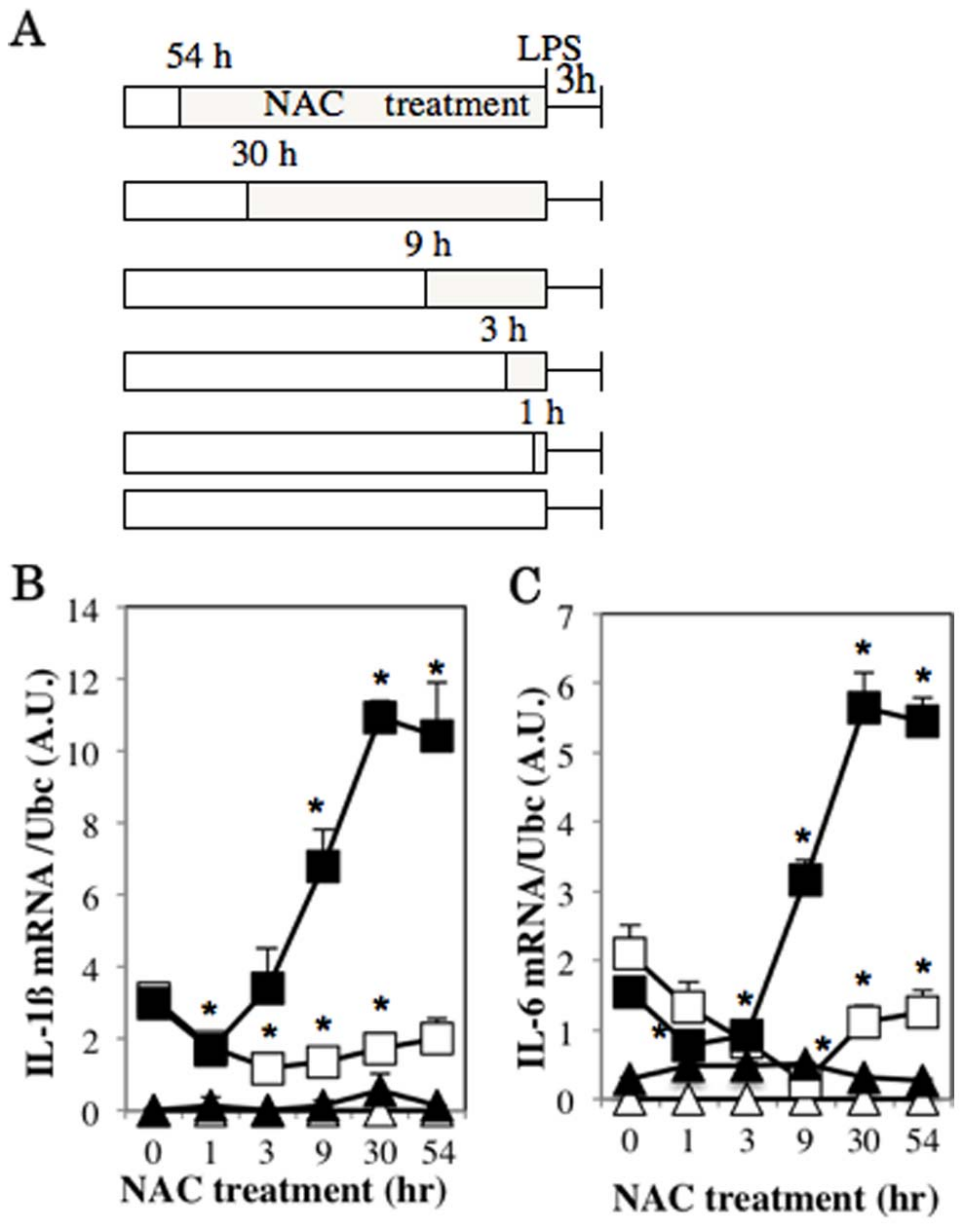
Time-dependent effects of low-dose NAC treatment on proinflammatory cytokine expressions in LPS-stimulated RAW264.7 cells. RAW264.7 cells were pretreated with 2(closed mark) or 20 mM (open mark) NAC following the indicated time schedule (A). The cells were further incubated in the presence (square) or absence (triangle) of 100 ng/ml LPS for 3 hours. Cellular IL-1β (B) and IL-6 (C) mRNA levels were detected by real time RT-PCR and normalized by Ubc mRNA levels (B, C). *p<0.05 versus time 0.

In order to confirm the previous finding that the short-time NAC treatment reduces expressions of proinflammatory cytokines, RAW264.7 cells were pretreated with 2 mM or 20 mM NAC for various time periods, followed by stimulation with LPS (Fig. 1A). In the result, 20 mM NAC pretreatment inhibited LPS-induced IL-1β and IL-6 mRNA expressions for as long as 9 hours (Fig. 1B). Similarly, short-time pretreatment with 2 mM NAC was inhibitory to these expressions. In contrast, however, when the cells were pretreated with 2 mM NAC for long-time (more than 9 hours), LPS-induced expressions of both IL-1β and IL-6 mRNAs were significantly enhanced (Fig. 1B, C).

We also examined the effect of long-time NAC treatment at 2 mM or 20 mM on IL-1β expression in LPS-stimulated human primary macrophages. Being similar to the findings of RAW264.7 cells, LPS-induced IL-1β mRNA expression was enhanced by low-dose (2 mM), but was inhibited by high-dose (20 mM) NAC treatment (Fig. S1).

### Activation of MAPKs and AKT, and Reduction of Phosphatase Expressions by NAC Treatment at a Low-concentration

**Figure 2:**
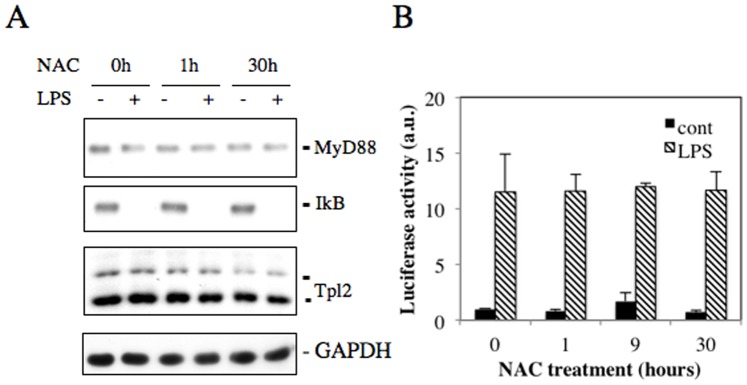
The effect of NAC treatment on TLR signal pathway in LPS-stimulated RAW264.7 cells. RAW264.7 cells were plated at 1.0×10^5^ cells/well, and cultured in DMEM containing 10% FCS with 2 mM NAC for 30 hours. 100 ng/ml LPS was added into the conditioned media, followed by further 3 hour incubation. A. The cell lysates were subjected to western blot analysis using anti-MyD88, cot/Tpl2, or IκBα antibody. B. The pNFκB-Luc plasmid was stably transfected into RAW264 cells. Cells were treated with 2 mM NAC for 1 hour, 9 hours or 30 hours, and stimulated with 100 ng/ml LPS for 3 hours. Luciferase activities in the cell lysates were normalized by the protein contents.

**Figure 3:**
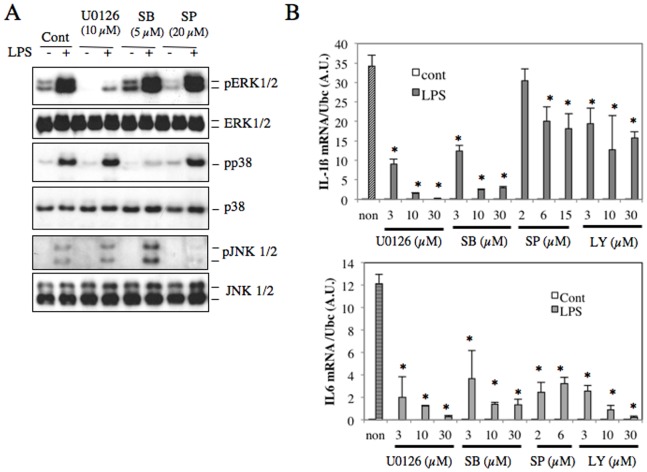
LPS-stimulated phosphorylation of MAPKs to induce proinflammatory cytokines. RAW264.7 cells were plated at 1.0×10^5^ cells/well and cultured in DMEM containing 10% FCS for 2 days. Cells were pretreated with various concentrations of U0126, SB203580 (SB), SP600125 (SP) or LY2904002 (LY) for 30 min, and stimulated with 100 ng/ml LPS for 20 min (A) or 3 hr (B). A. The cell lysates were subjected to western blot analysis using anti-pERK1/2, ERK1/2, pp38, p38, pJNK1/2, and JNK1/2. B. The gene expression of IL-1β and IL-6 was detected by real time RT-PCR. The mRNA levels were normalized by Ubc mRNA levels. *p<0.05 versus control.

**Figure 4:**
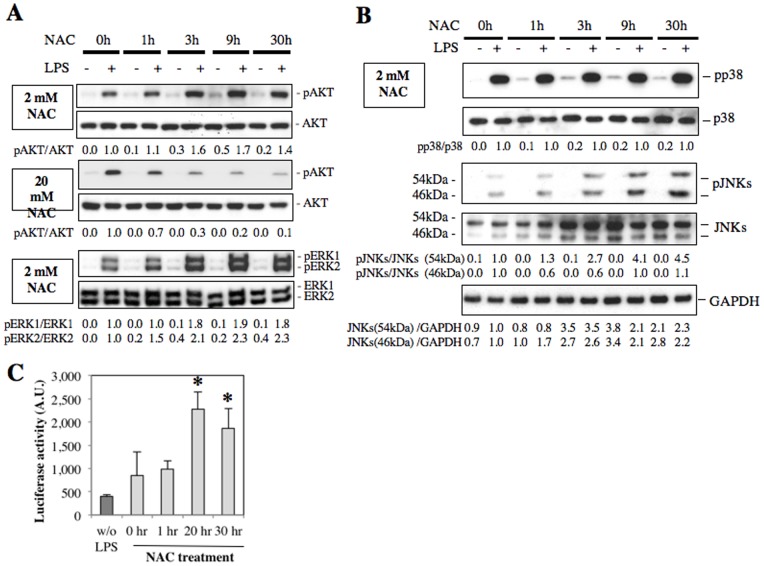
Effects of low-dose NAC treatment on phosphorylation of MAPKs and AKT in RAW264.7 cells. RAW264.7 cells were treated with 2/ml LPS was added into the conditioned media, and the cells were incubated for further 10 minutes (A, B). The cell lysates were subjected to western blot analysis using antibodies against pAKT, AKT, pERK1/2, ERK1/2 (A), pp38, p38, pJNK1/2, JNKs, or GAPDH (B). Ratios of AKT, ERK1/2 (A), p38 and JNK (B) phosphorylation relative to protein expression levels were photographically calculated using Image-J software (Image processing program). The relative phosphorylation levels in comparison to 0 h NAC treatment (set as 1.0) are shown in numbers. C. The pAP1-luc plasmid was stably transfected into RAW264.7 cells. The established cells were exposed to 2 mM NAC for 0 hour, 1 hour, 20 hours or 30 hours, and then the cells were stimulated with 100 ng/ml LPS for further 4 hours. Luciferase activities in the cell lysates were normalized by the protein contents. *p<0.05 versus control.

LPS/TLR4 signal-induced expressions of proinflammatory cytokines are mediated by various signaling molecules such as MyD88, Cot/Tpl2 and NF-κB [8], [27]. We found, however, that 2 mM NAC treatment did not affect activation or expression of these three molecules (Fig. 2). We next examined PI3K/AKT and MAPK signals, which are also activated by TLR4 signal [28], [29]. It was found that inhibitors of AKT and MAPKs reduced LPS-induced expressions of IL-1β and IL-6 in LPS-stimulated RAW264.7 cells (Fig. 3). As treatment with high-dose NAC has been reported to reduce IL-1β and IL-6 expressions in LPS-stimulated macrophages through the inhibition of AKT phosphorylation at S473 [13], [15], [17], we examined the effect of low-dose NAC on AKT phosphorylation in LPS-stimulated macrophages. We found that 2 mM NAC increased AKT phosphorylation in a time-dependent manner, whereas 20 mM NAC reduced its phosphorylation, suggesting that AKT phosphorylation may be involved in the regulation of proimflammatory cytokine expressions by NAC (Fig 4A).

We then examined the effect of 2 mM NAC treatment on the phosphorylation of MAPKs in LPS-stimulated RAW264.7 cells by western blot analyses. Treatment with 2 mM NAC enhanced phosphorylated JNKs and ERK1/2 levels after stimulation with LPS, but not p38, in time-dependent manners (Fig. 4A, B). Furthermore, phosphorylation of both p38 kinase and ERK1/2 was time-dependently increased by NAC treatment in RAW264.7 cells without LPS stimulation (Fig. 4A, B), possibly suggesting that reductions of some phosphatase activities for these MAPKs. On the other hand, NAC treatment increased both expression and phosphorylation levels of JNKs in LPS-stimulated RAW264.7 cells. The peak phosphorylation (30 hours) was induced later than the peak expression (3–9 hours) of JNKs (Fig. 4A, B).

As both mouse IL-1β and IL-6 gene promoters contain AP-1-binding sites [24], [30], we then examined the effect of 2 mM NAC treatment on AP-1 promoter activity using luciferase reporter assays. Long-time treatment with 2 mM NAC enhanced AP-1 promoter activity in LPS-stimulated RAW264.7 cells (Fig. 4D). As AP-1 is a known downstream target for MAPKs, it is presumed that the low-concentration NAC treatment increase proinflammatory cytokine expressions through MAPK-mediated AP-1 activation.

**Figure 5:**
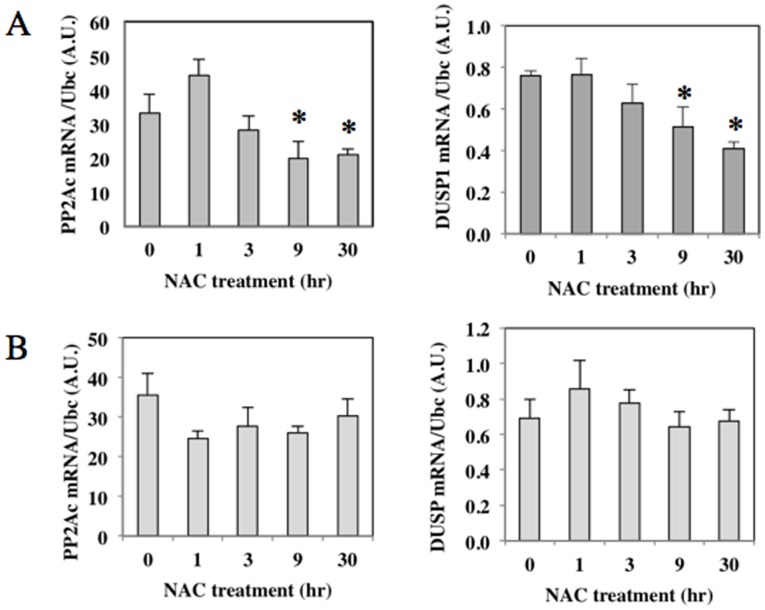
Reduction of phosphatase expressions by long-time NAC treatment. RAW264.7 cells were treated with 2(A) or 20 mM (B) NAC following the time schedule shown in Fig. 1A. Total RNA and cell lysates were extracted from the cells. The gene expression of PP2Ac and DUSP1 was detected by real time RT-PCR. The mRNA levels were normalized by Ubc mRNA levels. *p<0.05 versus time 0.

As for the MAPK activation mechanisms by low-dose NAC, we focused on two protein phosphatases, PP2Ac and DUSP1, because they have been reported to be expressed in macrophages [31]. DUSP1 dephosphorylates MAPKs including JNKs and ERK1/2, and PP2A dephosphorylates AKT and MAPKs [31]–[34]. We examined if NAC treatment affects the expression of these phosphatases in RAW264.7 cells, and found that 2 mM NAC treatment decreased expressions of both phosphatase mRNAs in time-dependent manners, whereas 20 mM NAC treatment did not (Fig. 5).

### Short-time NAC Treatment Inhibits Proinflammatory Cytokine Expressions through Induction of p53

**Figure 6:**
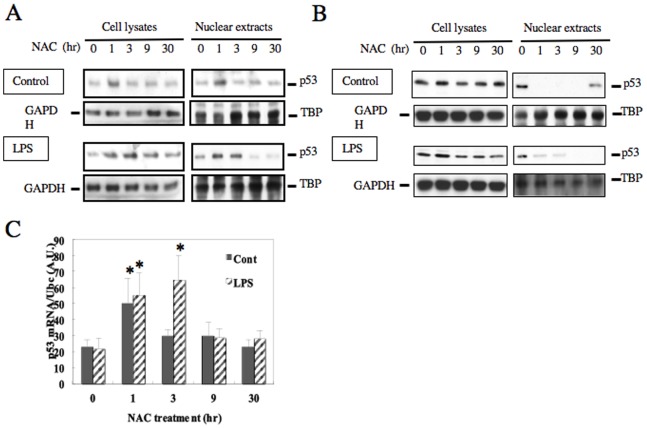
Effects of NAC treatment on p53 protein levels in the nuclear fraction of LPS-stimulated macrophages. A, B. RAW264.7 cells were pretreated with 2(A) or 20 mM (B) NAC for the indicated time. Cell lysates and nuclear extracts were prepared before (upper panels) and after (lower panels) stimulation with 100 ng/ml LPS for 3 hours, and then subjected to western blot analyses with anti-p53, anti-TBP, or anti-GAPDH antibody. C. RAW264.7 cells, pretreated with 2 mM NAC for the indicated time, were stimulated with 100 ng/ml LPS for further 3 hours, and then total RNA was extracted from the cells. p53 mRNA expression was analyzed by real time RT-PCR. The mRNA levels were normalized by Ubc mRNA levels. *p<0.05 versus time 0.

Although the long-time pretreatment with 2 mM NAC enhanced LPS-induced IL-1β and IL-6 mRNA expression, short-time (1 hr) pretreatment with the same NAC concentration decreased the induction of their expressions by 50% and 25%, respectively (Fig. 1B, C). In order to investigate the molecular mechanisms of this inhibitory effect, we examined p53 protein expression levels in RAW264.7 cells treated with NAC, as enhanced expression levels of IL-1β and IL-6 were recently reported in p53-null mice, suggesting that p53 is inhibitory to immune responses of macrophages [35]. When RAW264.7 cells were treated with 2 mM NAC, p53 protein levels in nuclear extracts and its mRNA expression increased within 1 hour of the pretreatment (Fig. 6A, C). On the other hand, 20 mM NAC treatment did not increase p53 levels in nuclear extracts from RAW264.7 cells stimulated with LPS (Fig. 6B), suggesting that p53 was not associated with reduction of IL-1β and IL-6 expressions by 20 mM NAC in the cells.

**Figure 7:**
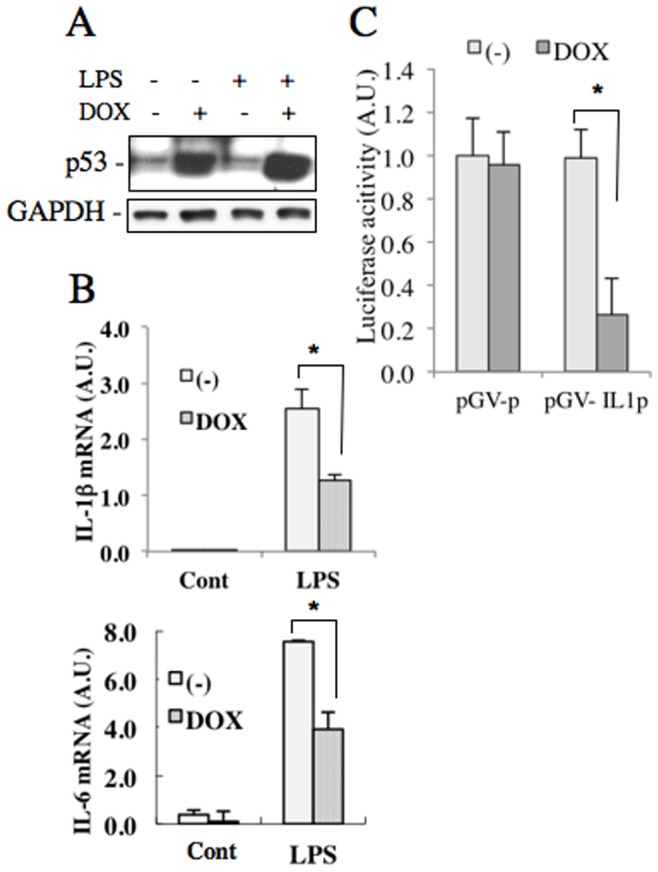
Reduction of interleukin expressions in LPS-stimulated RAW264.7 cells by over-expression of p53. RAW264.7 Tet-On stable cells were stably transfected with pTRE2Hyg-tp53, and treated with or without doxycycline. 48 hours later, the cells were treated with or without 100 ng/ml LPS for 3 hours, and the cell lysates and total RNA were extracted. A. Protein levels of p53 in the cell lysates were analyzed by western blot analysis. B. IL-1β and IL-6 mRNAs were detected by real time RT-PCR in total RNA from RAW264.7 Tet-On cells stably transfected with pTRE2Hyg-tp53. The mRNA levels were normalized by Ubc mRNA levels. (C) RAW264.7 Tet-On cells stably transfected with pTRE2Hyg-p53 were further transfected with pGV-IL1p. The resultant cells were treated with doxycycline for 48 hours. Luciferase activity in the cell lysates was measured and normalized by the protein content.

To verify that p53 regulates expressions of proinflammatory cytokines in LPS-stimulated macrophages, we prepared a Tet-On inducible expression system of p53 in RAW264.7 cells (Fig. 7A). We found that the doxycycline-induced overexpression of p53 reduced the mRNA expressions of IL-1β and IL-6 (Fig. 7B). Furthermore, IL-1β promoter assay showed that the doxycycline-induced expression of p53 reduced promoter activity of IL-1β (Fig. 7C). These observations indicated that the short-time NAC treatment at a low-concentration increased transcriptional activity of p53, which may be responsible for the reduced IL-1β expression.

On the other hand, high-dose NAC treatment reduced IL-1β and IL-6 expressions without increasing p53 expression (Fig. 1B, C and 6B). To investigate the molecular mechanisms of the reduced IL-1β and IL-6 expressions, we examined the effect of an AKT inhibitor, LY294002, on IL-1β and IL-6 mRNA expressions in RAW264.7 cells transfected with DOX-inducible p53 expression plasmid. In the result, removal of DOX from the media, that decreased p53 expression, increased IL-1β and IL-6 expressions in the cells (Fig. S2). Notably, inhibition of AKT activity reduced IL-1β and IL-6 expressions without the increase of p53 protein level (Fig. S2). These results suggested that high-dose NAC treatment decreased LPS-induced IL-1β and IL-6 expressions through the inhibition of AKT activation.

### The Effect of NAC Treatment on ROS Production from RAW264.7 Cells

**Figure 8:**
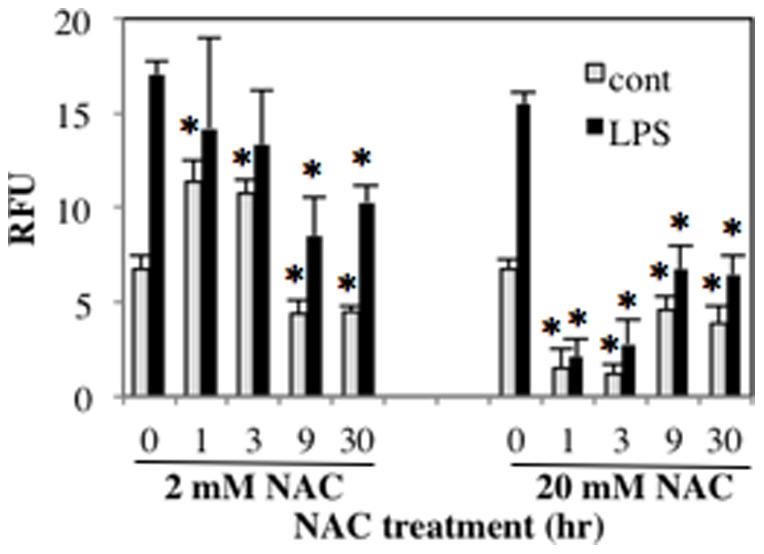
The effect of NAC treatment on ROS production in RAW264.7 cells. RAW264.7 cells were treated with 2 µM H_2_DCFDA-containing HBSS for 2 hours. The fluorescence emission of the cell suspensions was measured. *p<0.05 versus time 0, relatively. RFU, relative fluorescence units.

NAC is well known as an potent antioxidant. To examine the effect of NAC on LPS-induced ROS production, we measured ROS concentration in the culture supernatant of RAW264.7 cells stimulated with LPS (Fig. 8). Treatment with 20 mM NAC significantly inhibited ROS production from LPS-stimulated RAW264.7 cells. Similarly, 2 mM NAC treatment for more than 9 hours also decreased ROS concentration. However, 2 mM NAC treatment for 1 hour and 3 hours increased ROS concentration, suggesting that short-time treatment with low-dose NAC has no effect as an antioxidant.

## Discussion

In this study, we demonstrated that different concentrations of NAC caused opposite effects on the proinflammatory cytokine expressions in LPS-stimulated macrophages. This finding is supported by a previous report that different concentrations of NAC caused opposite effects on the ratio of reduced glutathione (GSH) to oxidized glutathione (GSSG) [36]. The GSH/GSSG balance is associated with regulation of IL-12 production [18]. A Th1 cytokine, IFN-γ, enhances IL-12 production in LPS-stimulated human alveolar macrophages (AM) and increase GSH/GSSG ratio in AM, whereas a Th2 cytokine, IL-4, reduces the IL-12 production and decreases the GSH/GSSG ratio [18]. On the other hand, NAC treatment at a low-concentration (2.5 mM) enhances the LPS-stimulated IL-12 production in both AM and a macrophage cell line, THP-1, and increases GSH/GSSG ratio in AM [18]. This report suggests that the GSH/GSSG balance, which is controlled by IFN-γ and IL-4, regulates IL-12 production in LPS-stimulated macrophages. Another previous report also showed that different doses of NAC exerted opposite effects on IL-12 expression in RAW264.7 cells without LPS-stimulation [36].

In our study, we found that low-dose NAC treatments for long-time increased expressions of IL-1β and IL-6 in LPS stimulated RAW264.7 cells, whereas high-dose NAC treatments decreased their expressions. Taken together with the previous reports mentioned above, our results suggested that the NAC-induced GSH/GSSG balance might regulate proinflammatory cytokine expressions in LPS-stimulated RAW264.7 cells. Furthermore, we also found that different concentrations of NAC induced opposite consequences in phosphorylation levels of several kinases, including ERK1/2, AKT and JNKs, suggesting that the GSH/GSSG balance might be associated with these kinase activities. Notably, we have shown that different concentrations of NAC cause opposite effects on phosphorylation of AKT, which was an upstream signal molecule of both NF-κB and AP-1 (Fig. S3). Moreover, our luciferase reporter gene assays showed that LPS-induced AP-1 promoter activity was enhanced by the low-dose NAC treatment. On the other hand, low-dose NAC treatment did not affect NF-κB promoter activity, being different from high-dose NAC treatment which is reported to be inhibitory to NF-kB promoter activity [13] (Fig. S3). These observations supported the idea that low-dose NAC stimulates AP-1 promoter activities of proinflammatory cytokine genes through activation of AKT as well as JNKs and ERK1/2 (Fig. S3).

Bacterial components such as LPS activate innate immune responses of macrophages [8]. The LPS/TLR4 signal is a potent inducer of IL-6 and IL-1β gene expressions in human and mouse macrophages through activation of various kinases including IKK, MAPKs, and AKT [10]. Here, our study using specific inhibitors of MAPKs suggested that ERK1/2 is an essential MAPK for the induction of IL-1β and IL-6. Activated ERK1/2 is dephosphorylated for inactivation by several phosphatases including PP2A, and DUSP1 [31], [37]. PP2A is a major serine/threonine protein phosphatase in various tissues and act as a tumor suppressor by inhibiting AKT and c-Myc phosphorylation [37]. Okadaic acid, a specific inhibitor of PP2A, induced expression of IL-1β in various cell types [38]. In our study we observed that long-time treatment with low-dose NAC decreased expression of PP2Ac and DUSP1, which may be responsible for the increased phosphorylation of ERK1/2 and AKT.

The important role of p53 as a tumor suppressor is the induction of cell cycle arrest of DNA-damaged cells to prevent proliferation of abnormal cells. ROS induce DNA-damage, which leads to increased expression of p53 [39]. While NAC is able to prevent ROS generation to decrease p53 expression, our result showed that low-dose NAC treatment for short-time did not decrease ROS generation (Fig. 8). On the contrary, low-dose NAC treatment for short-time increased expression of p53, which has been reported to decrease IL-1β and IL-6 expressions in Hela cells and T cells, respectively [40], [41]. We found that low-dose NAC pretreatments for long periods decreased IL-1β expression (Fig. 1B) and increased expression of p53 (Fig. 6), which was inhibitory to the promoter activity of IL-1β (Fig. 7). Expression of IL-6 is reported to be decreased by p53 through reduced promoter activity [40], [41]. Consistently, deficiency of p53 increases serum levels of IL-1β and IL-6 [35]. However, we could not find any consensus p53 binding sequences in IL-1β (−254 to +23) and IL-6 (−225 to +13) promoters [40], indicating that p53 does not directly bind to these promoter regions. Instead, both promoters contain binding motifs for CCAAT/enhancer binding protein β (C/EBPβ) An interaction of p53 with C/EBPβ is reported reciprocally inhibit transcriptional activities of both p53- and C/EBPβ-regulated genes [42]. Thus it may be suggested that the reduction of proinflammatory cytokine expressions by short-time NAC treatment at a low-concentration is caused by an indirect effect of p53 inhibiting transcriptional activity of C/EBPβ.

On the other hand, short-time treatment by high-dose NAC decreased IL-1β and IL-6 expressions without the increase of p53 in LPS-stimulated RAW264.7 cells. This reduction of the proinflammation cytokine expressions was assumed to be caused by decreased AKT phosphorylation (Fig. S2). In addition, we observed that NAC pretreatment for 3 hours enhanced phosphorylations of ERK1/2, JNKs and AKT in LPS-stimulated RAW264.7 cells without increasing IL-1β or IL-6 mRNA. We presume that this result may be associated with the increased p53 level, as p53 has been reported to decrease the IL-1β and IL-6 expressions in Hela cells and T cells, respectively [40], [41]. Since p53 decrease binding of c-Jun/c-Fos to AP-1 site to inhibit AP1-dependent promoter activity, p53 might negatively regulate the activation of AP-1, which is one of the downstream targets of AKT, in LPS-stimulated macrophages [43].

Stressful stimuli such as ROS and UV radiation activate MAPKs including JNKs and ERK1/2 to phosphorylate a serine residue in the transactivation domain of p53 protein [44]. This phosphorylation induces the activation and stabilization of p53 protein [44]. On the other hand, growth factors such as epidermal growth factor and hepatocyte growth factor also induce activation of the MAPKs such as ERK1/2 and p38 to induce cell proliferation, whereas they do not increase expression of p53 [45]–[47]. Conversely, activation of p53 by inhibition of murine double minute 2 is reported to enhance phosphorylation of ERK1/2 in reduction of osteosarcoma cells harboring wild-type p53 [48]. The report is consisted with our finding that 1 hour treatment with 2 mM NAC increased expression of p53 protein, and phosphorylation of ERK1/2 was induced after 3 hour treatment (Fig. 4A and 6A).

Most of the anti-oxidant functions of NAC are generally presumed to be through the regulation of intracellular GSH/GSSG balance [49]. Nevertheless, NAC is a thiol-containing compound that is able to interact with various molecules. The reaction of NAC with sulfenic acid derivatives in proteins can modify the activity of the proteins [49]. It is reported that NAC administration inhibits TNF-α signal to prevent the binding of TNF-α to its receptor, suggesting that NAC can react with proteins in extracellular environment [50]. Furthermore, NAC can also act as a pro-oxidant under special conditions. It has been reported that auto-oxidation processes of NAC can produce hydrogen peroxide in the presence of oxygen [51]. In our present study, short-time NAC treatment at low dose increased ROS concentration in RAW264.7 cells. NAC-induced apoptosis of transformed cell lines has been observed in a p53-dependent but GSH-independent manner. [52]. Taken together, our data have revealed that short-time treatment of macrophages with NAC at low-concentration did not reduce ROS generation and elevated p53 expression, which inhibited proinflammatory cytokine expressions.

NAC has been used in clinical trials of various infectious diseases, chronic bronchitis, hepatitis C virus infection, sepsis, and HIV infection [5]. NAC cleaves disulfide bounds in mucous glycoproteins to reduce viscosity, and this mucolytic action is thought to improve clinical conditions of chronic bronchopulmonary diseases such as chronic bronchitis [53]. As a matter of fact, NAC administration to chronic bronchitis is reported to decrease numbers of sick-leave days [54]. However, increased difficulty of sputum expectoration and cough severity were also reported to be greater in patients receiving NAC [55]. In addition, NAC administration did not affect virological responses in patients with chronic hepatitis C infection, and increased sequential organ failure assessment scores in patients with acute severe sepsis [21], [56]. A Trial of antiviral treatment with adjunctive NAC has shown that NAC increases GSH levels in blood and T cells, and improves stability of CD4-possitive cells in patients with HIV [57]. HIV is generally known to reduce cysteine and GSH levels in infected cells [58]. Therefore, these clinical trial results have indicated that NAC administration has not only immune-suppressive but also immuno-stimulatory effects. This idea is supported by our *in vitro* study that low-dose NAC treatment enhanced proinflammatory cytokines in activated macrophages.

## Supporting Information

Figure S1 (TIF)

Figure S2 (TIF)

Figure S3 (TIF)

## References

[1] Arakaki N, Kazi JA, Kazihara T, Ohnishi T, Daikuhara Y (1998) Hepatocyte growth factor/scatter factor activates the apoptosis signaling pathway by increasing caspase-3 activity in sarcoma 180 cells. Biochem. Biophys. Res. Commun. 245: 211–215. 10.1006/bbrc.1998.8397 9535810

[2] Thannickal VJ, Fanburg BL (2000) Reactive oxygen species in cell signaling. Am. J. Physiol. Lung Cell Mol. Physiol. 279: L1005–1028. 10.1152/ajplung.2000.279.6.L1005 11076791

[3] Kelly GS (1998) Clinical applications of N-acetylcysteine. Altern. Med. Rev. 3: 114–127. 9577247

[4] Scalley RD, Conner CS (1978) Acetaminophen poisoning: a case report of the use of acetylcysteine. Am. J. Hosp. Pharm. 35: 964–967. 677146

[5] Dodd S, Dean O, Copolov DL, Malhi GS, Berk M (2008) N-acetylcysteine for antioxidant therapy: pharmacology and clinical utility. Expert Opin. Biol. Ther. 8: 1955–1962. 10.1517/14728220802517901 18990082

[6] Ohnishi T, Bandow K, Kakimoto K, Machigashira M, Matsuyama T, et al (2009) Oxidative stress causes alveolar bone loss in metabolic syndrome model mice with type 2 diabetes. J. Periodontal Res. 44: 43–51. 10.1111/j.1600-0765.2007.01060.x 18973548

[7] Smith PD, Ochsenbauer-Jambor C, Smythies LE (2005) Intestinal macrophages: unique effector cells of the innate immune system. Immunol. Rev. 206: 149–159. 10.1111/j.0105-2896.2005.00288.x 16048547

[8] Raetz CR, Whitfield C (2002) Lipopolysaccharide endotoxins. Annu. Rev. Biochem. 71: 635–700. 10.1146/annurev.biochem.71.110601.135414 PMC2569852 12045108

[9] Kawai T, Akira S (2007) Signaling to NF-kappaB by Toll-like receptors. Trends Mol Med 13: 460–469. 18029230 10.1016/j.molmed.2007.09.002

[10] Brown J, Wang H, Hajishengallis GN, Martin M (2011) TLR-signaling networks: an integration of adaptor molecules, kinases, and cross-talk. J. Dent. Res. 90: 417–427. 10.1177/0022034510381264 PMC3075579 20940366

[11] Bandow K, Maeda A, Kakimoto K, Kusuyama J, Shamoto M, et al (2010) Molecular mechanisms of the inhibitory effect of lipopolysaccharide (LPS) on osteoblast differentiation. Biochem. Biophys. Res. Commun. 402: 755–761. 10.1016/j.bbrc.2010.10.103 21036155

[12] Haas A, Goebel W (1992) Microbial strategies to prevent oxygen-dependent killing by phagocytes. Free Radic. Res. Commun. 16: 137–157. 10.3109/10715769209049167 1601328

[13] Qi S, Xin Y, Guo Y, Diao Y, Kou X, et al (2012) Ampelopsin reduces endotoxic inflammation via repressing ROS-mediated activation of PI3K/Akt/NF-kappaB signaling pathways. Int. Immunopharmacol. 12: 278–287. 10.1016/j.intimp.2011.12.001 22193240

[14] Lin HY, Shen SC, Lin CW, Yang LY, Chen YC (2007) Baicalein inhibition of hydrogen peroxide-induced apoptosis via ROS-dependent heme oxygenase 1 gene expression. Biochim. Biophys. Acta. 1773: 1073–1086. 10.1016/j.bbamcr.2007.04.008 17532486

[15] Palacio JR, Markert UR, Martinez P (2011) Anti-inflammatory properties of N-acetylcysteine on lipopolysaccharide-activated macrophages. Inflamm. Res. 60: 695–704. 10.1007/s00011-011-0323-8 21424515

[16] Krifka S, Hiller KA, Spagnuolo G, Jewett A, Schmalz G, et al (2012) The influence of glutathione on redox regulation by antioxidant proteins and apoptosis in macrophages exposed to 2-hydroxyethyl methacrylate (HEMA). Biomaterials 33: 5177–5186. 22534037 10.1016/j.biomaterials.2012.04.013

[17] Hsu HY, Wen MH (2002) Lipopolysaccharide-mediated reactive oxygen species and signal transduction in the regulation of interleukin-1 gene expression. J. Biol. Chem. 277: 22131–22139. 10.1074/jbc.M111883200 11940570

[18] Dobashi K, Aihara M, Araki T, Shimizu Y, Utsugi M, et al (2001) Regulation of LPS induced IL-12 production by IFN-gamma and IL-4 through intracellular glutathione status in human alveolar macrophages. Clin Exp Immunol 124: 290–296. 11422207 10.1046/j.1365-2249.2001.01535.x PMC1906042

[19] Sadowska AM, Verbraecken J, Darquennes K, De Backer WA (2006) Role of N-acetylcysteine in the management of COPD. Int. J. Chron. Obstruct. Pulmon. Dis. 1: 425–434. 10.2147/copd.2006.1.4.425 PMC2707813 18044098

[20] Dean O, Giorlando F, Berk M (2011) N-acetylcysteine in psychiatry: current therapeutic evidence and potential mechanisms of action. J. Psychiatry Neurosci. 36: 78–86. 10.1503/jpn.100057 PMC3044191 21118657

[21] Spapen HD, Diltoer MW, Nguyen DN, Hendrickx I, Huyghens LP (2005) Effects of N-acetylcysteine on microalbuminuria and organ failure in acute severe sepsis: results of a pilot study. Chest 127: 1413–1419. 15821223 10.1378/chest.127.4.1413

[22] Kane LP, Shapiro VS, Stokoe D, Weiss A (1999) Induction of NF-kappaB by the Akt/PKB kinase. Curr Biol 9: 601–604. 10359702 10.1016/s0960-9822(99)80265-6

[23] Gustin JA, Korgaonkar CK, Pincheira R, Li Q, Donner DB (2006) Akt regulates basal and induced processing of NF-kappaB2 (p100) to p52. J Biol Chem 281: 16473–16481. 16613850 10.1074/jbc.M507373200

[24] Cahill CM, Rogers JT (2008) Interleukin (IL) 1beta induction of IL-6 is mediated by a novel phosphatidylinositol 3-kinase-dependent AKT/IkappaB kinase alpha pathway targeting activator protein-1. J. Biol. Chem. 283: 25900–25912. 10.1074/jbc.M707692200 PMC2533786 18515365

[25] Matsuguchi T, Musikacharoen T, Johnson TR, Kraft AS, Yoshikai Y (2001) A novel mitogen-activated protein kinase phosphatase is an important negative regulator of lipopolysaccharide-mediated c-Jun N-terminal kinase activation in mouse macrophage cell lines. Mol. Cell. Biol. 21: 6999–7009. 10.1128/MCB.21.20.6999-7009.2001 PMC99875 11564882

[26] Matsuguchi T, Chiba N, Bandow K, Kakimoto K, Masuda A, et al (2009) JNK activity is essential for Atf4 expression and late-stage osteoblast differentiation. J. Bone Miner. Res. 24: 398–410. 10.1359/jbmr.081107 19016586

[27] Ohnishi T, Okamoto A, Kakimoto K, Bandow K, Chiba N, et al (2010) Involvement of Cot/Tp12 in bone loss during periodontitis. J. Dent. Res. 89: 192–197. 10.1177/0022034509353405 20089988

[28] Scherle PA, Jones EA, Favata MF, Daulerio AJ, Covington MB, et al (1998) Inhibition of MAP kinase kinase prevents cytokine and prostaglandin E2 production in lipopolysaccharide-stimulated monocytes. J. Immunol. 161: 5681–5686. 9820549

[29] Kang JS, Yoon YD, Lee KH, Park SK, Kim HM (2004) Costunolide inhibits interleukin-1beta expression by down-regulation of AP-1 and MAPK activity in LPS-stimulated RAW 264.7 cells. Biochem. Biophys. Res. Commun. 313: 171–177. 10.1016/j.bbrc.2003.11.109 14672714

[30] Perez RL, Ritzenthaler JD, Roman J (1999) Transcriptional regulation of the interleukin-1beta promoter via fibrinogen engagement of the CD18 integrin receptor. Am. J. Respir. Cell Mol. Biol. 20: 1059–1066. 10.1165/ajrcmb.20.5.3281 10226077

[31] Patterson KI, Brummer T, O’Brien PM, Daly RJ (2009) Dual-specificity phosphatases: critical regulators with diverse cellular targets. Biochem. J. 418: 475–489. 10.1042/bj20082234 19228121

[32] Lang R, Hammer M, Mages J (2006) DUSP meet immunology: dual specificity MAPK phosphatases in control of the inflammatory response. J. Immunol. 177: 7497–7504. 10.4049/jimmunol.177.11.7497 17114416

[33] Traore K, Sharma R, Thimmulappa RK, Watson WH, Biswal S, et al (2008) Redox-regulation of Erk1/2-directed phosphatase by reactive oxygen species: role in signaling TPA-induced growth arrest in ML-1 cells. J. Cell. Physiol. 216: 276–285. 10.1002/jcp.21403 PMC2587147 18270969

[34] Liao Y, Hung MC (2010) Physiological regulation of Akt activity and stability. Am. J. Transl. Res. 2: 19–42. PMC2826820 20182580

[35] Zheng SJ, Lamhamedi-Cherradi SE, Wang P, Xu L, Chen YH (2005) Tumor suppressor p53 inhibits autoimmune inflammation and macrophage function. Diabetes 54: 1423–1428. 15855329 10.2337/diabetes.54.5.1423

[36] Alam K, Ghousunnissa S, Nair S, Valluri VL, Mukhopadhyay S (2010) Glutathione-redox balance regulates c-rel-driven IL-12 production in macrophages: possible implications in antituberculosis immunotherapy. J Immunol 184: 2918–2929. 20164428 10.4049/jimmunol.0900439

[37] Zhang Q, Claret FX (2012) Phosphatases: the new brakes for cancer development? Enzyme Res. 2012: 659649. 10.1155/2012/659649 PMC3206369 22121480

[38] Lee CH, Chen JC, Hsiang CY, Wu SL, Wu HC, et al (2007) Berberine suppresses inflammatory agents-induced interleukin-1beta and tumor necrosis factor-alpha productions via the inhibition of IkappaB degradation in human lung cells. Pharmacol. Res. 56: 193–201. 10.1016/j.phrs.2007.06.003 17681786

[39] Vurusaner B, Poli G, Basaga H (2012) Tumor suppressor genes and ROS: complex networks of interactions. Free Radic Biol Med 52: 7–18. 22019631 10.1016/j.freeradbiomed.2011.09.035

[40] Santhanam U, Ray A, Sehgal PB (1991) Repression of the interleukin 6 gene promoter by p53 and the retinoblastoma susceptibility gene product. Proc. Natl. Acad. Sci. U S A 88: 7605–7609. 10.1073/pnas.88.17.7605 PMC52350 1652755

[41] Zhang S, Zheng M, Kibe R, Huang Y, Marrero L, et al (2011) Trp53 negatively regulates autoimmunity via the STAT3-Th17 axis. FASEB J. 25: 2387–2398. 10.1096/fj.10-175299 PMC3114529 21471252

[42] Schneider-Merck T, Pohnke Y, Kempf R, Christian M, Brosens JJ, et al (2006) Physical interaction and mutual transrepression between CCAAT/enhancer-binding protein beta and the p53 tumor suppressor. J Biol Chem 281: 269–278. 16227626 10.1074/jbc.M503459200

[43] Tu SP, Chi AL, Ai W, Takaishi S, Dubeykovskaya Z, et al (2009) p53 inhibition of AP1-dependent TFF2 expression induces apoptosis and inhibits cell migration in gastric cancer cells. Am J Physiol Gastrointest Liver Physiol 297: G385–396. 19541923 10.1152/ajpgi.90620.2008 PMC2724087

[44] Wu GS (2004) The functional interactions between the p53 and MAPK signaling pathways. Cancer Biol. Ther. 3: 156–161. 10.4161/cbt.3.2.614 14764989

[45] Minden A, Lin A, McMahon M, Lange-Carter C, Derijard B, et al (1994) Differential activation of ERK and JNK mitogen-activated protein kinases by Raf-1 and MEKK. Science 266: 1719–1723. 7992057 10.1126/science.7992057

[46] Leotoing L, Manin M, Monte D, Baron S, Communal Y, et al (2007) Crosstalk between androgen receptor and epidermal growth factor receptor-signalling pathways: a molecular switch for epithelial cell differentiation. J. Mol. Endocrinol. 39: 151–162. 10.1677/JME-07-0021 17693613

[47] Matteucci E, Modora S, Simone M, Desiderio MA (2003) Hepatocyte growth factor induces apoptosis through the extrinsic pathway in hepatoma cells: favouring role of hypoxia-inducible factor-1 deficiency. Oncogene 22: 4062–4073. 12821940 10.1038/sj.onc.1206519

[48] Lee SY, Shin SJ, Kim HS (2013) ERK1/2 activation mediated by the nutlin-3-induced mitochondrial translocation of p53. Int. J. Oncol. 42: 1027–1035. 10.3892/ijo.2013.1764 23314357

[49] Parasassi T, Brunelli R, Costa G, De Spirito M, Krasnowska E, et al (2010) Thiol redox transitions in cell signaling: a lesson from N-acetylcysteine. Scientific World Journal 10: 1192–1202. 20602078 10.1100/tsw.2010.104 PMC5763934

[50] Hayakawa M, Miyashita H, Sakamoto I, Kitagawa M, Tanaka H, et al (2003) Evidence that reactive oxygen species do not mediate NF-kappaB activation. EMBO J. 22: 3356–3366. 10.1093/emboj/cdg332 PMC165656 12839997

[51] Tuttle S, Horan AM, Koch CJ, Held K, Manevich Y, et al (1998) Radiation-sensitive tyrosine phosphorylation of cellular proteins: sensitive to changes in GSH content induced by pretreatment with N-acetyl-L-cysteine or L-buthionine-S,R-sulfoximine. Int. J. Radiat. Oncol. Biol. Phys. 42: 833–838. 10.1016/s0360-3016(98)00331-9 9845106

[52] Liu M, Pelling JC, Ju J, Chu E, Brash DE (1998) Antioxidant action via p53-mediated apoptosis. Cancer Res. 58: 1723–1729. 9563490

[53] Grandjean EM, Berthet P, Ruffmann R, Leuenberger P (2000) Efficacy of oral long-term N-acetylcysteine in chronic bronchopulmonary disease: a meta-analysis of published double-blind, placebo-controlled clinical trials. Clin Ther 22: 209–221. 10743980 10.1016/S0149-2918(00)88479-9

[54] Rasmussen JB, Glennow C (1988) Reduction in days of illness after long-term treatment with N-acetylcysteine controlled-release tablets in patients with chronic bronchitis. Eur Respir J 1: 351–355. 3294038

[55] Jackson IM, Barnes J, Cooksey P (1984) Efficacy and tolerability of oral acetylcysteine (Fabrol) in chronic bronchitis: a double-blind placebo controlled study. J Int Med Res 12: 198–206. 6376210 10.1177/030006058401200312

[56] Grant PR, Black A, Garcia N, Prieto J, Garson JA (2000) Combination therapy with interferon-alpha plus N-acetyl cysteine for chronic hepatitis C: a placebo controlled double-blind multicentre study. J Med Virol 61: 439–442. 10897061 10.1002/1096-9071(200008)61:4<439::aid-jmv5>3.0.co;2-l

[57] Spada C, Treitinger A, Reis M, Masokawa IY, Verdi JC, et al (2002) The effect of N-acetylcysteine supplementation upon viral load, CD4, CD8, total lymphocyte count and hematocrit in individuals undergoing antiretroviral treatment. Clin Chem Lab Med 40: 452–455. 12113286 10.1515/CCLM.2002.077

[58] Nakamura H, Masutani H, Yodoi J (2002) Redox imbalance and its control in HIV infection. Antioxid Redox Signal 4: 455–464. 12215212 10.1089/15230860260196245

