# Supplementary figures and images for: Long-Time Treatment by Low-Dose N-Acetyl-L-Cysteine Enhances Proinflammatory Cytokine Expressions in LPS-Stimulated Macrophages
Source: PLoS One. 2014 Feb 4;9(2):e87229. doi: 10.1371/journal.pone.0087229 (PMC3913600; doi:10.1371/journal.pone.0087229)

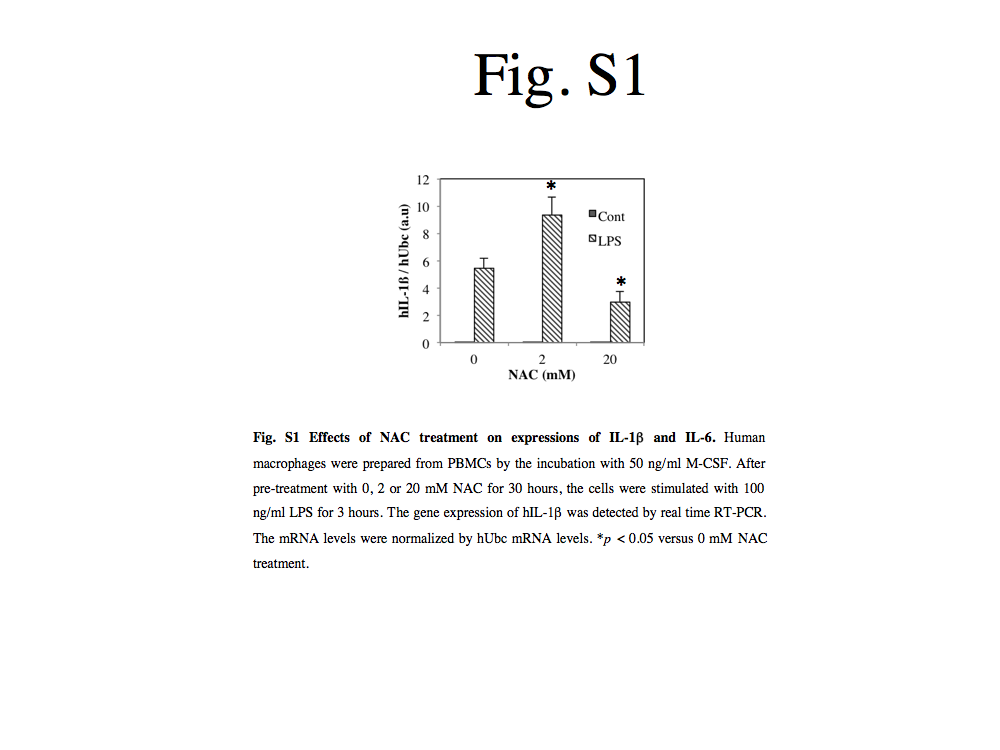

Supplement: Figure S1 — (TIF) [file pone.0087229.s001.tif]

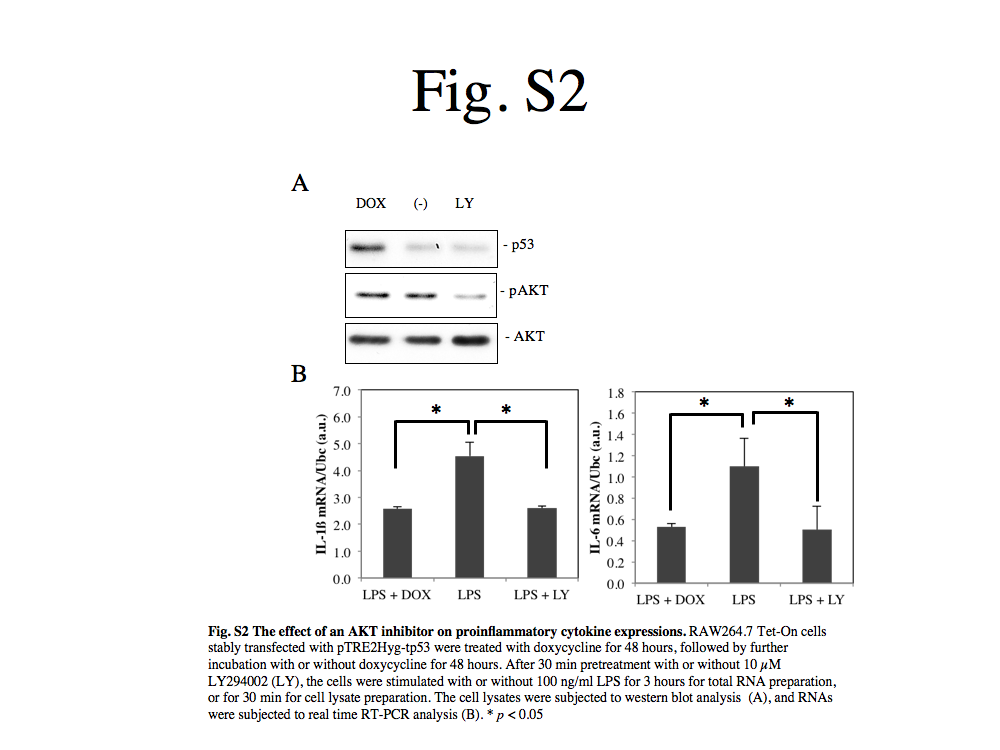

Supplement: Figure S2 — (TIF) [file pone.0087229.s002.tif]

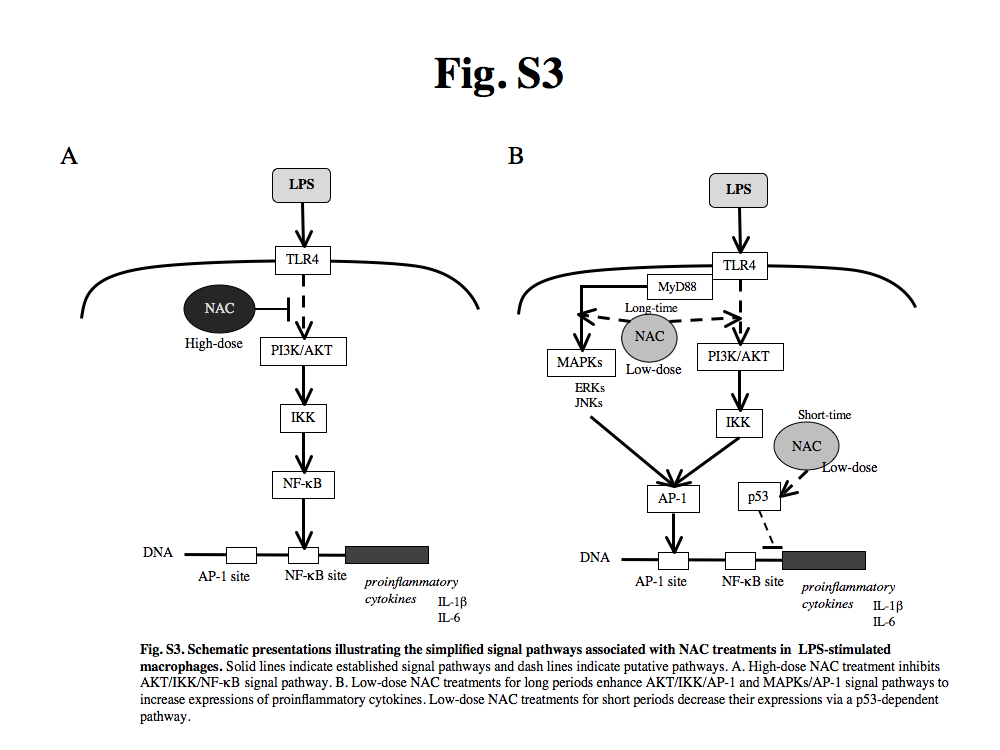

Supplement: Figure S3 — (TIF) [file pone.0087229.s003.tif]
